# Inflammatory Molecule Elaboration in Secondhand Smoke (SHS)-Induced or Conditional RAGE Transgenic Modeling of Chronic Rhinosinusitis (CRS)

**DOI:** 10.3390/cimb47090740

**Published:** 2025-09-10

**Authors:** Logan Ponder, Ryan Kinney, Ankita Chatterjee, Kristina Vu, Harishma Sidhu, Neha Patel, Tejus Desai, Daniel L. Orr, Juan A. Arroyo, Paul R. Reynolds

**Affiliations:** 1 College of Dental Medicine, Roseman University of Health Sciences, South Jordan, UT 84095, USA; 2 School of Dental Medicine, University of Nevada Las Vegas, Las Vegas, NV 89102, USA; 3 Anesthesiology, Jurisprudence, Oral and Maxillofacial Surgery, University of Nevada, Las Vegas, NV 89102, USA; 4 Department of Cell Biology and Physiology, Brigham Young University, Provo, UT 84602, USA

**Keywords:** inflammation, sinusitis, RAGE, cytokines

## Abstract

Chronic rhinosinusitis (CRS) is characterized by sinonasal inflammation, mucus overproduction, and edematous mucosal tissue. This inflammatory condition is characterized by mucosal thickening, nasal obstruction, facial pain or pressure, hyposmia, and nasal discharge. The aim of this research was to clarify a potential role for the receptor for advanced glycation end-products (RAGE) in mouse nasoantral epithelium in perpetuating pro-inflammatory cytokine elaboration similarly expressed by CRS patients. Specifically, wild-type (WT) mice and transgenic (TG) mice overexpressing RAGE in sinonasal epithelium (RAGE TG mice) were maintained in room air or subjected to secondhand smoke exposure using a nose-only delivery system (Scireq Scientific, Montreal, QC, Canada) for five days per week over a 30-day period. Histological analysis was performed using staining for RAGE. Tissue lysates were analyzed for pro-inflammatory cytokines. We observed increased RAGE expression in sinus tissue following SHS exposure and in sinuses from RAGE TG mice in the absence of SHS. We also discovered elevated T helper (Th)1 products (TNF-α, IL-1β, IFN-γ) and Th2/Th17 (IL-5, IL-13, IL-17A) cytokine abundance in SHS-exposed WT and SHS-exposed RTG tissues compared to room air controls. These findings highlight the pivotal role of RAGE signaling in the exacerbation of inflammatory processes, particularly in the context of chronic inflammation induced by smoke exposure. The study expands our understanding of the RAGE signaling axis as a key contributor to the progression of smoke-related lung and sinonasal pathologies. Targeting RAGE-mediated pathways could represent a novel therapeutic strategy to mitigate the progression of chronic sinusitis associated with smoke exposure.

## 1. Introduction

Chronic rhinosinusitis (CRS) is a multifactorial inflammatory disease affecting the paranasal sinuses for more than 12 weeks, characterized by persistent mucosal inflammation, epithelial barrier dysfunction, and cytokine-driven immune dysregulation. The disease is subtyped into CRS with nasal polyps (CRSwNP) and CRS without nasal polyps (CRSsNP), each marked by distinct immune signatures that reflect underlying Th cell polarization [1]. While Th2-dominant inflammation, driven by cytokines such as IL-4, IL-5, and IL-13, is well established in eosinophilic CRSwNP, recent work has highlighted the importance of Th1 (e.g., IFN-γ) and Th17 (e.g., IL-17A, IL-22) pathways in CRSsNP and in neutrophilic or mixed inflammatory phenotypes of CRSwNP [2,3]. In fact, pediatric CRSwNP demonstrates mixed Th2/Th17 patterns, while CRSsNP more consistently exhibits Th1/Th17 signatures, suggesting that age and disease subtype significantly influence the cytokine milieu [3]. Recent findings indicate that Th17 cytokines, particularly IL-17 and IL-22, directly disrupt the sinonasal epithelial barrier by altering tight junctions and increasing permeability, providing a mechanistic link between inflammation and mucosal compromise [4]. These barrier defects may facilitate persistent exposure to microbial and environmental triggers, reinforcing chronic inflammation. Interestingly, there is substantial evidence of interplay and cross-regulation between the Th2 and Th17 pathways. In a study of Chinese CRSwNP patients, IL-4 and IL-13 suppressed Th17 cytokine expression, while IL-17 and TGF-β1 enhanced Th2 cytokines, suggesting that Th2 and Th17 responses may reciprocally modulate each other depending on the local immune environment [5].

**Figure 1:**
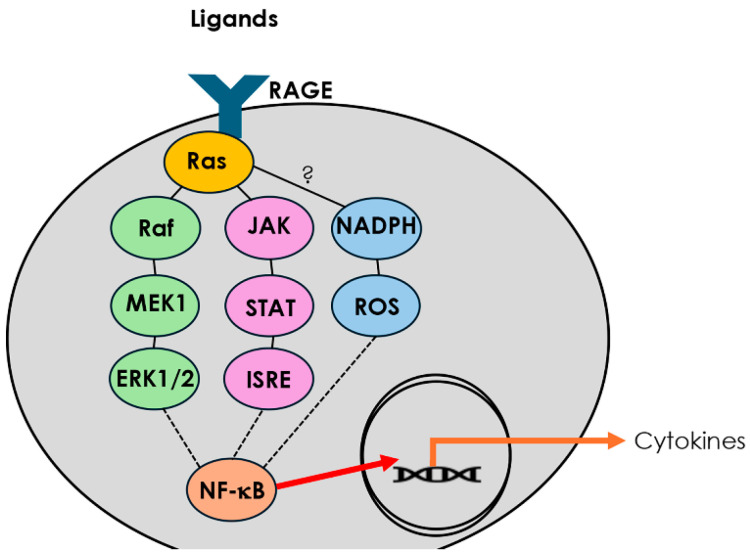
**RAGE signaling pathways leading to cytokine release.** Ligand binding to the receptor for advanced glycation end products (RAGE) activates Ras and initiates multiple intracellular signaling cascades. The Ras/Raf/MEK/ERK1/2 pathway, together with JAK/STAT/ISRE signaling, converge on NF-κB activation. In parallel, RAGE stimulation engages NADPH oxidase, generating reactive oxygen species (ROS) that further enhance NF-κB activity. Activated NF-κB translocates to the nucleus, driving transcription of pro-inflammatory cytokine genes.

Secondhand smoke (SHS) remains a pervasive environmental toxin and an established contributor to airway disease. Even brief exposures to SHS can impair epithelial barrier function, increase mucociliary dysfunction, and promote chronic inflammation in both upper and lower airways [6]. SHS contains a mixture of reactive aldehydes, particulate matter, and advanced glycation end-products (AGEs) capable of activating pattern recognition receptors such as the receptor for advanced glycation end-products (RAGE). RAGE is a multiligand receptor belonging to the immunoglobulin superfamily and is highly expressed in pulmonary tissues, particularly in alveolar and epithelial cells [7]. Upon engagement with its ligands—including HMGB1, S100 proteins, and AGEs—RAGE causes the activation of diverse signaling intermediates that coordinate sustained NF-κB activation, driving chronic production of inflammatory mediators and perpetuating tissue damage (Figure 1). Multiple studies have identified upregulation of RAGE in response to tobacco smoke exposure in diverse tissue types, including lung, vasculature, placenta, and upper-airway tissue, reinforcing its role as a key sensor of noxious environmental stimuli [7,8,9]. In the context of CRS, RAGE activation may serve as an upstream driver of multiple inflammatory phenotypes. Engagement of RAGE in the sinonasal epithelium can promote the secretion of pro-inflammatory cytokines such as IL-1β and TNF-α and has been associated with both eosinophilic (Th2) and neutrophilic (Th1/Th17) inflammatory signatures depending on the local microenvironment and genetic background.

In our previous work, we demonstrated that exposure to SHS significantly upregulates RAGE expression in both human and murine sinonasal tissues. Using a novel TG mouse model with doxycycline-inducible RAGE expression in sinus epithelium, we showed that RAGE overexpression alone, without smoke exposure, was sufficient to induce hallmark features of chronic sinusitis, including epithelial apoptosis (cleaved caspase-3), elevated levels of pro-inflammatory cytokines, and matrix remodeling enzymes MMP-9 and MMP-13 [10]. Given RAGE’s known role in amplifying NF-κB signaling, we have now hypothesized that its activation may also exacerbate Th1/Th2/Th17 axis dysregulation, shifting cytokine balance toward a chronic inflammatory state. Indeed, RAGE activation has been associated with elevated IL-17A and IL-5 in human nasal polyps [11], and S100/calgranulin ligands have been shown to amplify Th17-mediated epithelial damage [12]. To further dissect this axis, we now examine cytokine secretion patterns in murine sinus tissue following either 30 days of SHS exposure or RAGE overexpression. We focused specifically on signature Th1 (IFN-γ), Th2 (IL-4, IL-5, IL-13), and Th17 (IL-17A, IL-22) cytokines, aiming to understand how RAGE signaling intersects with adaptive immune polarization in the sinonasal microenvironment.

## 2. Materials and Methods

### 2.1. Transgenic Mice and Experimental Exposures

Double TG K14-rtTA/tetO-RAGE mice (designated RTG) were used to selectively induce RAGE expression in sinonasal epithelial cells upon doxycycline administration, as described in detail in [10]. Animals were housed in pathogen-free conditions under a 12 h light/dark cycle with access to standard chow and water ad libitum. Mice were randomly assigned to one of three treatment groups (n = 8 per group): (1) wild-type (WT) mice exposed to room air (WT RA), (2) WT mice exposed to secondhand smoke (WT SHS), (3) RTG mice exposed to RA (RTG). SHS exposure was conducted using a nose-only InExpose system (Scireq, Montreal, QC, Canada), delivering sidestream smoke from 3R4F research cigarettes over 30 days as previously described [8] and similarly restrained mice exposed to room air (RA) were used as controls. On the date of necropsy, biopsies that contained sinus cavities were inflation-fixed with 4% paraformaldehyde for histology or were used to isolate total protein. Mice were housed and utilized in accordance with an animal use protocol (#24-0203) (Approval date: 15 March 2024) approved by the Institutional Animal Care and Use Committee (IACUC) at Brigham Young University and carried out in accordance with the prevailing regulations.

### 2.2. Tissue Collection and Histological Processing

Mice were sacrificed and biopsies that contained sinus cavities were inflation-fixed with 4% paraformaldehyde for histology. Immunohistochemical localization of RAGE (R&D Systems, Minneapolis, MN, USA; Cat #mAb1179, 1:500) was performed on sections as previously outlined. These immunohistochemical stains involved at least eight images evaluated from each mouse (n = 8 per group). A no-primary-antibody control served as a negative control and no immunoreactivity was observed. Stained sections were imaged using an Olympus BX51 microscope (Center Valley, PA, USA) and Olympus CellSens Standard 3.1.

### 2.3. Cytokine Screening by Antibody-Based Dot Blot

Sinonasal epithelium was removed from murine nasal septa and sinus cavities by dissection and washed with HEPES-DMEM containing 1% penicillin–streptomycin to remove blood and other debris. Protein isolation was performed by homogenization with RIPA buffer containing protease inhibitors (Fisher Scientific, Waltham, MA, USA). Total protein was quantified using a BCA Protein Assay Kit (Fisher Scientific). For each treatment group, protein from four mice was pooled to yield 125 μg of total protein per blot. Mouse Cytokine Array Panel A (R&D Systems, Minneapolis, MN, USA) was used to screen for cytokines associated with Th1, Th2, and Th17 pathways. Blots were incubated overnight with samples, then with biotin-conjugated antibodies, followed by streptavidin-conjugated IRDye^®^ 800CW (LI-COR Biosciences, Lincoln, NE, USA). Fluorescent signals were captured using an Odyssey DLx imaging system and analyzed using ImageJ software, Version 1.54h. Representative dot blot quantifications of pro-inflammatory cytokines (TNF-α, IL-1β, IFN-γ) and type 2/17 cytokines (IL-5, IL-13, IL-17A) are presented with the pixel intensity for each target normalized to positive control spots on the membrane.

### 2.4. Statistical Analysis

Data are presented as means ±SE. One-way ANOVA was used to compare protein expression levels among treatment groups, followed by Tukey’s post hoc test to determine pairwise group differences. A *p*-value of <0.05 was considered statistically significant. Statistical analysis was conducted using GraphPad Prism 8.0 software.

## 3. Results

### 3.1. RAGE Overexpression and SHS Exposure Induce Sinonasal Epithelial Remodeling

**Figure 2:**
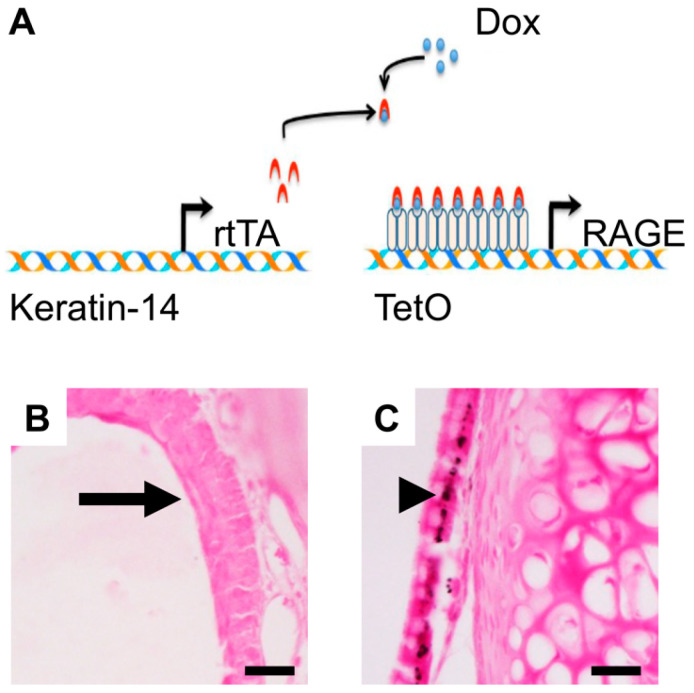
**Histological assessment of sinonasal epithelium.** (**A**) Schematic of RAGE overexpression in doxycycline-induced K14-rtTA/tetO-RAGE TG mice. Immunohistochemical staining was used to confirm RAGE expression in sinonasal epithelial tissue of RTG mice. (**B**) Staining for RAGE revealed no immunoreactivity in sinus sections from WT + RA group ((**B**), arrow) or in RTG mice in the absence of Dox [10]. (**C**) Marked expression of RAGE was detected in sinonasal epithelial tissue from RTG mice ((**C**), arrowhead). Images are representative of 4 randomized fields per animal, with n = 8 mice per group. Scale bars = 50 μm.

The functionality of the doxycycline-inducible RAGE overexpression system was confirmed in K14-rtTA/tetO-RAGE (RTG) mice (Figure 2A). Histological examination in our previous manuscript [10] revealed epithelial thickening and goblet cell hyperplasia in sinonasal tissue from both RTG and wild-type mice exposed to SHS (WT SHS) compared to wild-type room air controls (WT RA). Representative sections stained for RAGE revealed no immunoreactivity in sinus epithelium from control mice (Figure 2B, arrow) and string RAGE immunostaining in sinus epithelium from RTG mice (Figure 2C, arrowhead).

### 3.2. SHS and RAGE Drive Upregulation of Th1 and Pro-Inflammatory Cytokines

**Figure 3:**
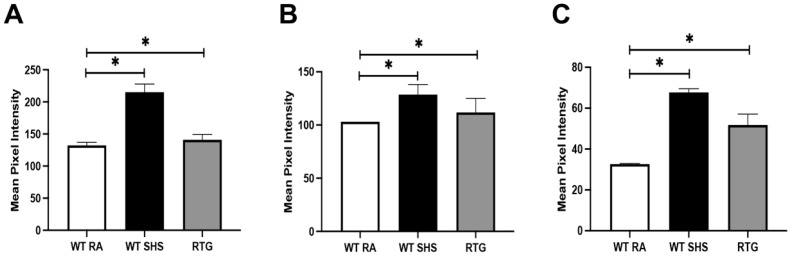
Upregulation of Th1 and pro-inflammatory cytokines in mouse sinus tissue following SHS exposure or RAGE overexpression. Dot blot analysis was used to quantify Th1-related cytokines from sinus protein lysates across three experimental groups (WT RA, WT SHS, and RTG). (**A**) TNF-α levels were significantly increased in both WT SHS and RTG mice compared to WT RA controls (*p* < 0.05). (**B**) IL-1β was significantly elevated in WT SHS mice and RTG mice compared to RA controls (*p* < 0.05). (**C**) IFN-γ expression was significantly higher in both SHS (*p* < 0.01) and RTG (*p* < 0.05) mice compared to controls. Importantly, RAGE upregulation in RTG mice was sufficient to induce significant cytokine secretion comparable to SHS-exposed mice. There was no significant difference in the levels of secreted cytokines when comparing WT + RA and RTG + RA. Densitometric analysis is based on relative pixel intensity normalized to loading controls. n = 8 mice per group, and * indicates statistical significance (*p* < 0.05).

Dot blot analysis demonstrated significantly elevated levels of TNF-α in both the WT SHS and RTG groups compared to WT RA (*p* < 0.05; Figure 3A). Specifically, the TNF-α mean pixel intensity was ~200 in SHS and ~145 in RTG mice versus ~130 in controls. IL-1β levels were also significantly increased in the SHS group (*p* < 0.05 vs. WT RA), with a significant elevation also observed in RTG mice compared to controls (Figure 3B). Similarly, IFN-γ expression was significantly higher in both SHS (*p* < 0.01) and RTG mice (*p* < 0.05) compared to controls (Figure 3C), consistent with Th1 pathway activation.

### 3.3. SHS and RAGE Promote Type 2 and Type 17 Cytokine Responses

**Figure 4:**
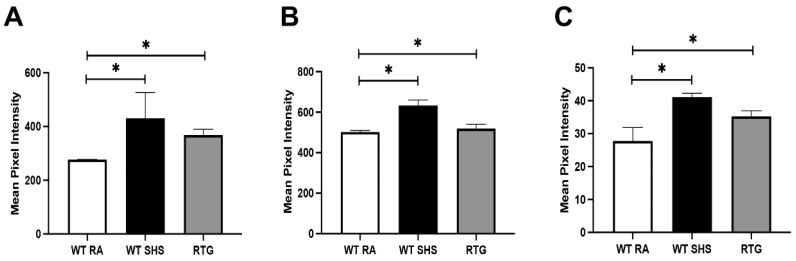
**Induction of Th2 and Th17 cytokines by SHS exposure or epithelial-specific RAGE upregulation.** (**A**) IL-5 expression was significantly increased in sinus tissue lysates from both WT SHS and RTG mice versus WT RA controls (*p* < 0.05), consistent with Th2-mediated inflammation. (**B**) IL-13 levels were similarly elevated in the SHS and RTG groups (*p* < 0.05). (**C**) IL-17A, a signature Th17 cytokine, showed significantly enhanced expression in both experimental groups relative to controls (*p* < 0.05). Importantly, RAGE upregulation in RTG mice was sufficient to induce significant cytokine secretion comparable to SHS-exposed mice. There was no significant difference in the levels of secreted cytokines when comparing WT + RA and RTG + RA. Cytokine signals were quantified using dot blot densitometry and reflect average signal intensities normalized to housekeeping controls. The data are the means ± SE from n = 8 animals per group, and * denotes significance at *p* < 0.05.

We next examined Th2- and Th17-associated cytokines. IL-5 levels were significantly increased in both the WT SHS and RTG groups relative to WT RA (*p* < 0.05 for both comparisons; Figure 4A), with mean pixel intensities of ~425 (SHS), ~385 (RTG), and ~260 (RA). IL-13 showed a similar pattern, with significantly elevated expression in SHS and RTG mice compared to controls (*p* < 0.05; Figure 4B). Notably, IL-17A levels were also significantly increased in both experimental groups versus WT RA mice (*p* < 0.05 for both; Figure 4C), reinforcing the activation of Th17-associated inflammatory pathways.

Together, these findings indicate that both SHS and epithelial-specific RAGE overexpression were sufficient to promote a pro-inflammatory sinonasal microenvironment, characterized by increased Th1, Th2, and Th17 cytokine expression. While SHS produced a somewhat stronger cytokine response overall, the RTG model recapitulates many of the same immunologic changes in the absence of SHS, supporting RAGE as a key mediator of CRS-like inflammation.

## 4. Discussion

This study expands upon our prior work implicating RAGE as a central mediator of sinonasal inflammation by demonstrating that both SHS exposure and epithelial-specific RAGE overexpression result in a broad upregulation of pro-inflammatory and Th cell-associated cytokines in murine sinonasal tissue. These findings provide mechanistic insight into how environmental and molecular insults converge to disrupt sinonasal immune homeostasis and promote CRS-like pathology. Consistent with prior observations in both CRSwNP and CRSsNP patients, we observed significant elevations in key Th1 cytokines—TNF-α, IL-1β, and IFN-γ—following SHS and RAGE activation [2,13,14]. TNF-α and IL-1β are well-established initiators of epithelial damage, mucosal permeability, and neutrophil recruitment, while IFN-γ enhances antigen presentation and amplifies type 1 immunity. Our model supports a synergistic role for RAGE and SHS in sustaining a pro-inflammatory milieu dominated by Th1 effector signals. Strikingly, both RAGE overexpression and SHS exposure also induced robust increases in Th2 cytokines IL-5 and IL-13—hallmarks of eosinophilic inflammation in CRSwNP [5,11,14]. IL-5 promotes eosinophil survival and recruitment, while IL-13 drives goblet cell metaplasia and mucus hypersecretion—features clearly seen in our histologic analysis. The concurrent rise in Th2 cytokines in the RTG group suggests that RAGE may act upstream of canonical type 2 signaling and could contribute to the Th2 polarization observed in subsets of CRS patients. Most notably, both experimental conditions induced significant upregulation of IL-17A, a signature Th17 cytokine linked to neutrophilic inflammation, epithelial barrier disruption, and steroid resistance in CRS [3,4,15]. IL-17A has been shown to compromise tight junction integrity and promote persistent barrier leakage in CRS epithelium, contributing to microbial translocation and chronic inflammation. The consistent IL-17A elevation in both the RTG and SHS groups implicates RAGE in the orchestration of type 17 responses, potentially through activation of NF-κB and downstream IL-6 and IL-23 production.

Interestingly, our data also align with recent findings describing overlapping Th1, Th2, and Th17 responses in CRS tissues, particularly in mixed or neutrophilic endotypes [16,17,18]. These mixed cytokine signatures may reflect disease plasticity or distinct temporal stages of CRS progression, with RAGE functioning as a critical node in shaping early inflammatory polarization. Regional and ethnic variation in CRS cytokine patterns has been well documented, with some populations exhibiting Th1/Th17 dominance (e.g., in China), while others show strong Th2/eosinophilic profiles (e.g., Europe, Oceania) [2,12]. The fact that both SHS and RAGE drove upregulation of all three major Th axes in our study suggests that this model captures key aspects of these divergent endotypes and may be useful for evaluating therapeutic strategies in CRS subpopulations. Moreover, the detection of type 1, type 2, and type 17 cytokines in RTG mice in the absence of environmental smoke implicates RAGE as a potent driver of inflammation independent of exogenous insults. This aligns with studies identifying high RAGE expression in inflamed mucosa from CRS and nasal polyp patients, particularly in neutrophilic or non-eosinophilic subtypes [19,20].

It is important to note that the current investigation evaluated the phenotypic outcomes of SHS exposure and RAGE upregulation in the context of CRS. While these differential modalities culminate in phenotypic characteristics observed in CRS progression, they may not completely substitute for all causes of the disease. As such, future endeavors should involve the development of additional CRS models so comparison studies that include SHS or RAGE upregulation can be more precisely evaluated. Our findings clearly support the emerging consensus that RAGE plays a central role in mediating cellular responses to tobacco-related insults across diverse tissue types. Previous studies have demonstrated that RAGE is upregulated in the lung, placenta, vasculature, and sinonasal epithelium following exposure to cigarette smoke or secondhand smoke, implicating it in a wide range of inflammatory and fibrotic processes. These data highlight RAGE as a ubiquitous sensor of environmental danger signals and suggest its relevance beyond the upper airway. However, we recognize that our inducible K14-rtTA/TetO-RAGE transgenic model, while robust and tractable, has inherent limitations. Although keratin-14 is expressed by basal cells in the sinonasal epithelium, its expression is not strictly confined to the upper airway, and off-target RAGE overexpression in skin, oral mucosa, or other K14-positive epithelia is possible. As such, further characterization of this model is needed to comprehensively map the spatial distribution of RAGE expression and to assess localized versus systemic inflammatory changes. Future studies should also evaluate specific cellular populations for proliferative, apoptotic, and immunologic phenotypes, along with full-spectrum histopathologic analysis. Nonetheless, the present study provides a crucial step toward defining the mechanistic role of RAGE in chronic sinonasal inflammation and offers a foundation for investigating how epithelial–environmental interactions contribute to CRS pathogenesis.

In summary, these findings support the hypothesis that RAGE acts as a convergent upstream regulator of multiple T-helper cell pathways in CRS pathogenesis. We observed potent inflammatory outcomes in tissues exposed to SHS. While RAGE targeting was sufficient to induce inflammation, expression of markers was lower in RAGE KO mice compared to mice exposed to SHS. Consequently, RAGE targeting may ameliorate inflammatory characteristics but is unlikely to model the complete pathogenesis of CRS alone. By enhancing responsiveness to environmental stimuli and perpetuating inflammatory cytokine loops, RAGE may help drive both initiation and chronicity of sinonasal disease. Therapeutically, targeting RAGE or its downstream mediators may offer a strategy to disrupt this cycle and favor mucosal immune balance.

## Data Availability

All data are presented within the article. Data and other materials are available from the corresponding author on reasonable request.
